# Supplementary material for: Comparison of Venous Blood Gas and Biochemical Parameters in Sunda Pangolin (Manis javanica) and Chinese Pangolin (Manis pentadactyla) before and after Isoflurane Anesthesia
Source: Animals (Basel). 2023 Mar 25;13(7):1162. doi: 10.3390/ani13071162 (PMC10093139; doi:10.3390/ani13071162)
Supplement: Supplementary file 1 [file animals-13-01162-s001.zip › animals-2264496-supplementary.pdf]

Supplementary Material

Supplementary Tables

Table S1. Weight (kg), Total length (cm), and Tail length (cm) data of Chinese pangolins (n_{adult} = 28).

Animal ID	sex	weight	Total length	Tail length
MP-1	♀	4.17	67	28
MP-2	♀	5.96	83	35
MP-3	♀	3.15	71	28
MP-4	♀	3.53	70	28
MP-5	♀	3.69	71	30
MP-6	♀	3.49	63.5	25
MP-7	♀	3.12	67	28
MP-8	♀	3.49	69	29
MP-9	♀	3.1	67	27.5
MP-10	♀	3.43	72	30.5
MP-11	♀	3.26	69	28
MP-12	♀	3.29	68	30
MP-13	♀	3.25	71	29
MP-14	♀	3.43	69	28
MP-15	♂	5.94	76	32
MP-16	♂	4.36	74	31
MP-17	♂	5.74	92	35
MP-18	♂	3.97	74	30.5
MP-19	♂	4.32	76.5	32
MP-20	♂	3.93	71	30
MP-21	♂	4.31	74	31
MP-22	♂	3.29	66	26
MP-23	♂	4.2	77.4	27.6
MP-24	♂	3.6	72	30.6
MP-25	♂	4.7	81.9	30
MP-26	♂	4.36	74	30
MP-27	♂	4.32	76.5	32
MP-28	♂	4.31	75	31

Table S2. Weight (kg), Total length (cm), and Tail length (cm) data of Sunda pangolins (n_{adult} = 26).

Animal ID	sex	weight	Total length	Tail length
MJ-1	♀	4.39	90	40.5
MJ -2	♀	4.62	89	40
MJ -3	♀	5.27	91	42
MJ -4	♀	5.48	91	41
MJ -5	♀	5.93	91	40
MJ -6	♀	4.82	87	40
MJ -7	♀	4.84	89	40
MJ -8	♀	4.61	88	40
MJ -9	♀	3.71	82	36
MJ -10	♀	4.29	90.1	44.3
MJ -11	♀	5.2	90	41
MJ -12	♀	5.3	93	42
MJ -13	♀	4.7	90	40
MJ -14	♀	5.626	97	44.5
MJ -15	♂	5.39	93	42
MJ -16	♂	7.72	107	48
MJ -17	♂	8.87	106	48
MJ -18	♂	7	99	46
MJ -19	♂	8.47	100	45
MJ -20	♂	8.71	110	53
MJ -21	♂	8.64	106	49
MJ -22	♂	6.66	96	44
MJ -23	♂	5	90	40
MJ -24	♂	5.43	119	54
MJ -25	♂	5.4	121	54.5
MJ -26	♂	6	105	49

Table S3. Venous blood gas and biochemical analyses in awake (T1) of Chinese pangolins (n_{adult} = 28).

Animal ID		MP -1	MP - -2	MP - -3	MP - 4	MP -5	MP -6	MP -7	MP -8	MP -9	MP -10	MP -11	MP -12	MP -13	MP -14	MP -15	MP -16	MP -17	MP -18	MP -19	MP -20	MP -21	MP -22	MP -23	MP -24	MP -25	MP -26	MP -27	MP -28
Body temperature	℃	32.8	32.4	32.4	32.6	32	33.6	33	33.8	32.2	31.9	33	32.5	32.6	32.8	32.2	32.3	33	32.3	33.6	33.2	32.3	33	33.2	33.1	32.6	32.7	32.8	32.8
H^+	10^{-7} mol/L	6.03	3.55	6.31	5.89	3.98	5.01	4.37	3.31	4.07	5.50	4.47	4.79	4.17	5.25	7.08	5.13	6.46	6.03	5.50	6.76	6.61	5.75	4.57	5.13	5.25	6.17	6.31	5.01
pH	—	7.22	7.45	7.2	7.23	7.4	7.3	7.36	7.48	7.39	7.26	7.35	7.32	7.38	7.28	7.15	7.29	7.19	7.22	7.26	7.17	7.18	7.24	7.34	7.29	7.28	7.21	7.2	7.3
PO_2	mmHg	102	149	25	116	99	104	52	165	67	45	98	90	89	74	72	35	103	47	73	29	114	105	90	125	111	111	65	107
PCO_2	mmHg	74	58	95	55	75	58	62	45	44	84	66	64	60	78	75	86	58	78	80	90	71	62	75	50	70	69	80	71
pHt	—	7.28	7.52	7.26	7.29	7.47	7.35	7.42	7.53	7.46	7.33	7.41	7.38	7.44	7.34	7.21	7.36	7.24	7.28	7.31	7.22	7.24	7.29	7.39	7.34	7.34	7.27	7.26	7.36
PO_2t	mmHg	80	124	18	92	73	85	39	148	48	31	77	68	67	56	52	25	81	34	58	22	88	83	71	103	87	87	49	84
PCO_2t	mmHg	62	47	78	45	60	50	52	39	36	67	55	53	49	65	61	70	49	64	69	76	58	52	64	42	58	57	67	59
TCO_2	mmol/L	32.6	42.1	40	24.7	48.8	30.3	36.9	34.9	28	40.3	38.4	35	37.3	39.1	28.4	44	24	34.3	38.4	35.6	28.7	28.5	42.8	25.5	35	29.7	33.8	37.1
Beecf	mmol/L	2.6	16.3	9.1	-4.6	21.7	2.1	9.6	10	1.6	0.6	10.8	6.9	10.4	10	-2.8	14.8	-6	4.2	8.8	4.3	-1.9	-0.8	14.7	-2.6	6.2	-0.3	3.3	8.5
BE(B)	mmol/L	-0.9	12.5	5.6	-5.8	15.1	0.8	6.2	8.4	1	5.6	7.1	3.7	7.1	5	-5.6	9.9	-7.4	0.5	4.7	-0.5	-4.7	-3	10.3	-3.4	3.3	-3.2	-0.1	5
$HCO3^-$	mmol/L	30.3	40.3	37.1	23	46.5	28.5	35	33.5	26.6	37.7	36.4	33	35.5	36.7	26.1	41.4	22.2	31.9	35.9	32.8	26.5	26.6	40.5	24	32.9	27.6	31.3	34.9
$HCO3^-$ (std)	mmol/L	24.2	34.7	27.5	20.4	36.7	25.5	29.2	31.6	25.5	28.3	30.4	27.7	30.4	28.6	20.2	31.3	19.1	24.3	28.4	22	21.2	22.5	32.9	22.3	27.5	22.4	24.5	28.8
K^+	mmol/L	5.5	5	3.7	4.6	5.5	4.5	5.1	4.7	4.9	5.3	5	6.3	4.8	5	6.5	4.8	4.5	4.4	4.7	4.5	4.9	5.3	4.4	5.4	4.3	5.4	5.7	4.7
Na^+	mmol/L	151	149	152	151	145	155	142	145	147	144	148	147	141	148	149	144	146	147	146	151	143	144	146	145	145	150	144	139
$iCa2^+$	mmol/L	1.26	1.34	1.24	1.28	1.12	1.34	1.19	1.35	1.19	1.25	1.25	1.23	1.26	1.45	1.22	1.54	1.26	1.2	1.24	1.21	1.27	1.27	1.32	1.22	1.2	1.23	1.3	1.23
Glu	mmol/L	2.6	3.9	4.4	7.2	5.9	4.1	5.3	4.7	6.3	4.3	4.3	5.3	4.8	5.9	4.9	5.1	6.8	5.1	5	4.7	4.9	5.3	4.9	6	4.8	4.8	4.9	6.1
Lac	mmol/L	9.1	5.1	8.5	>15	7.8	12.1	5.8	6.4	7.7	3.7	4.4	6.3	3.2	4.2	>15	4.5	14.4	9.1	10.3	13.6	12.4	>15	3.6	13.1	5.1	14.6	12.2	5.9
nCa^{2+}	mmol/L	1.17	1.37	1.14	1.19	1.12	1.29	1.17	1.39	1.19	1.18	1.22	1.19	1.25	1.38	1.1	1.47	1.16	1.11	1.17	1.1	1.16	1.19	1.29	1.17	1.14	1.14	1.2	1.18
Hct	%	62	58	>65	64	64	>65	61	56	58	59	58	61	60	>65	59	51	61	57	54	62	63	63	52	53	49	64	52	54
SO2	%	97	99	31	98	98	97	85	100	93	73	97	96	97	93	89	59	96	73	92	38	97	97	96	98	98	97	87	98
THBC	g/L	211	197	—	218	218	—	207	190	197	201	197	207	204	221	201	173	207	194	184	211	214	214	177	180	167	218	177	184

Table S4. Venous blood gas and biochemical analyses in isoflurane anaesthesia (T2) of Chinese pangolins (n_{adult} = 28).

Animal ID		MP -1	MP - -2	MP - -3	MP - 4	MP -5	MP -6	MP -7	MP -8	MP -9	MP -10	MP -11	MP -12	MP -13	MP -14	MP -15	MP -16	MP -17	MP -18	MP -19	MP -20	MP -21	MP -22	MP -23	MP -24	MP -25	MP -26	MP -27	MP -28
Body temperature	°C	34.2	33	33.1	34.5	32.7	34.2	33.8	33.8	34.7	32.6	33.1	33.4	33	33.5	32.8	32.2	33.5	32.5	33.6	32.8	32.9	33.7	33.1	34.2	32.3	32.7	33.4	33
H^+	10^{-7} mol/L	7.24	4.90	4.57	6.03	4.27	5.89	4.90	5.89	4.27	5.62	4.27	5.89	5.13	4.47	10.72	5.01	7.41	6.31	4.79	5.37	6.92	6.61	4.79	4.37	4.79	6.61	5.01	4.90
pH	—	7.14	7.31	7.34	7.22	7.37	7.23	7.31	7.23	7.37	7.25	7.37	7.23	7.29	7.35	6.97	7.3	7.13	7.2	7.32	7.27	7.16	7.18	7.32	7.36	7.32	7.18	7.3	7.31
PO_2	mmHg	245	275	244	231	349	307	219	405	327	418	213	400	438	191	323	74	64	379	377	456	323	335	69	337	213	285	267	86
PCO_2	mmHg	102	81	74	75	80	78	69	102	58	85	60	82	81	73	109	83	81	89	82	86	89	90	83	71	74	90	84	72
pHt	—	7.18	7.37	7.4	7.25	7.43	7.27	7.36	7.27	7.4	7.31	7.43	7.28	7.35	7.4	7.02	7.37	7.18	7.26	7.37	7.33	7.21	7.22	7.38	7.4	7.39	7.24	7.35	7.37
PO_2t	mmHg	232	257	226	219	327	294	204	387	316	393	194	380	415	173	303	54	50	355	359	431	303	319	53	323	191	265	250	67
PCO_2t	mmHg	90	68	62	67	66	68	60	89	52	70	51	70	68	63	91	67	70	73	71	72	74	78	70	63	60	75	72	60
TCO_2	mmol/L	37.8	43.3	42.2	33	48.7	35.1	36.8	45.8	35.3	39.9	36.5	36.8	41.4	42.5	28.4	43.3	29.4	37.5	44.7	42.1	34.4	36.4	45.3	42.3	40.4	36.4	43.9	38.5
Beecf	mmol/L	5.7	14.5	14.1	3	20.9	5.1	8.4	15.1	8.2	10.1	9.4	6.7	12.3	14.7	-6.7	14.4	-2.3	6.8	16.1	12.6	3	5.2	16.7	14.7	12	5.2	14.9	10
BE(B)	mmol/L	0.5	9.3	9.4	-0.4	14.9	1.2	5	9.1	5.9	5.9	6.4	2.8	8.2	10.3	-11	10.7	-5.3	2.8	12.2	8.5	-1.2	1.6	12.3	11.2	9.2	0.9	11.2	7.1
$HCO3^-$	mmol/L	34.7	40.8	39.9	30.7	46.2	32.7	34.7	42.7	33.5	37.3	34.7	34.3	38.9	40.3	25.1	40.8	26.9	34.8	42.2	39.5	31.7	33.6	42.8	40.1	38.1	33.6	41.3	36.3
$HCO3^-$ (std)	mmol/L	25.4	32.3	32.3	24.7	36.6	25.9	28.9	32.1	29.6	29.6	32	27.2	31.4	33	16.4	33.2	20.3	27.2	34.5	31.6	24.1	26.2	34.3	33.7	32.2	25.7	33.7	30.4
K^+	mmol/L	3.7	3.8	3.6	3.4	3.8	3.8	4.2	4.2	4	4.7	4.1	4.2	4.1	3.8	3.2	3.9	3.7	3.6	3.9	3.1	3.9	4.1	3.8	4.1	4	3.7	3.9	4
Na^+	mmol/L	149	148	152	148	141	149	141	146	145	140	146	143	139	148	146	144	147	144	145	145	142	139	145	143	144	146	140	139
$iCa2^+$	mmol/L	1.27	1.32	1.21	1.23	1.07	1.32	1.15	1.38	1.16	1.24	1.16	1.17	1.24	1.44	1.14	1.51	1.25	1.16	1.24	1.16	1.21	1.26	1.28	1.18	1.18	1.15	1.18	1.24
Glu	mmol/L	7.3	4.6	4.8	7.2	6.6	5.8	6.3	5.9	5.5	5.2	4.2	7	4.8	5.7	7.4	5.2	7.5	4.8	4.7	4.6	5.8	7.4	5.7	5.4	4.8	5.5	6.4	5.4
Lac	mmol/L	9.2	3.5	1.6	11.7	6.3	9.6	5.9	4.8	3.7	1.3	4.5	4.4	3.2	1.6	14.9	3.2	11	4.8	2.6	5.5	7.5	10.5	2.1	2.7	1.7	7.9	1.7	3.4
nCa^{2+}	mmol/L	1.14	1.27	1.18	1.14	1.06	1.23	1.11	1.29	1.15	1.17	1.15	1.09	1.19	1.41	0.96	1.45	1.12	1.07	1.2	1.1	1.1	1.15	1.24	1.16	1.14	1.05	1.13	1.2
Hct	%	57	57	58	58	56	60	55	53	49	50	58	53	48	53	58	41	54	49	41	45	58	47	45	44	38	55	40	43
SO2	%	100	100	100	100	100	100	100	100	100	100	100	100	100	100	100	93	84	100	100	100	100	100	92	100	100	100	100	96
THBC	g/L	194	194	197	197	190	204	187	180	167	170	197	180	163	180	197	139	184	167	139	153	197	160	153	150	128	187	136	146

Table S5. Venous blood gas and biochemical analyses in awake (T1) of Sunda pangolins (n_{adult} = 26).

**Animal ID**		MJ-1	MJ-2	MJ-3	MJ-4	MJ-5	MJ-6	MJ-7	MJ-8	MJ-9	MJ-10	MJ-11	MJ-12	MJ-13	MJ-14	MJ-15	MJ-16	MJ-17	MJ-18	MJ-19	MJ-20	MJ-21	MJ-22	MJ-23	MJ-24	MJ-25	MJ-26
Body temperature	℃	33.3	33.3	33.4	33.9	33.1	33.1	33.3	33.4	33	33.6	33.2	33	33.1	33.1	33.9	33.1	33.1	32.7	32.4	32.9	33	33.1	32.9	33	32.5	33.3
H^+	10^{-7} mol/L	4.47	4.07	5.37	4.17	4.17	5.89	4.47	6.92	4.68	4.57	4.37	3.39	4.37	5.13	4.17	3.02	5.01	5.75	6.03	7.94	3.72	7.59	6.92	3.47	8.13	4.79
pH	—	7.35	7.39	7.27	7.38	7.38	7.23	7.35	7.16	7.33	7.34	7.36	7.47	7.36	7.29	7.38	7.52	7.3	7.24	7.22	7.1	7.43	7.12	7.16	7.46	7.09	7.32
PO_2	mmHg	77	63	55	102	126	74	23	37	115	144	139	137	132	96	62	130	95	126	85	90	128	50	95	136	56	129
PCO_2	mmHg	52	62	52	56	44	80	100	109	53	40	49	44	36	59	64	46	45	43	81	96	51	101	80	43	96	46
pHt	—	7.4	7.44	7.32	7.43	7.44	7.28	7.4	7.21	7.39	7.39	7.41	7.53	7.42	7.34	7.43	7.58	7.35	7.3	7.28	7.15	7.49	7.17	7.21	7.52	7.15	7.37
PO_2t	mmHg	60	49	43	85	104	57	18	29	93	125	118	115	110	76	50	108	75	102	63	70	105	38	74	113	41	108
PCO_2t	mmHg	44	53	44	49	37	67	85	93	44	34	41	37	30	50	56	39	38	36	66	80	43	85	67	36	79	39
TCO_2	mmol/L	30.3	39.4	25.5	34.8	27.4	36	58.3	42.2	29.5	22.8	29.2	33.4	21.4	30.2	39.9	39	23.5	19.7	35.7	32.7	35.5	35.9	31	31.9	32	25.1
Beecf	mmol/L	3.1	12.5	-3	8	0.9	5.9	29.6	10.2	2	-4.2	2.3	8.3	-5.1	1.8	12.8	14.7	-4.3	-9	5.5	0.1	9.6	3.5	-0.2	6.8	-0.7	-2.4
BE(B)	mmol/L	1.8	9.1	-3.6	5.7	0.4	2.1	20.4	4.2	0.6	-3.9	1.2	7.4	-4.4	0.2	8.6	12.7	-4.5	-8.8	1.7	-3.7	7.4	-1.6	-3.2	5.8	-4.5	-2.8
$HCO3^-$	mmol/L	28.7	37.5	23.9	33.1	26	33.5	55.2	38.9	27.9	21.6	27.7	32	20.3	28.4	37.9	37.6	22.1	18.4	33.2	29.8	33.9	32.8	28.5	30.6	29.1	23.7
$HCO3^-$ (std)	mmol/L	26.2	31.8	21.7	29.4	25.3	26.3	38.3	26.5	25.4	21.9	25.9	30.7	21.5	25.1	31.3	34.9	21.4	18.1	26.1	21.9	30.7	22.5	22.3	29.5	20.6	22.8
K^+	mmol/L	4.9	5.1	6	5.1	5.7	5.5	4.3	5.5	5.8	5.1	5.7	4.6	5.2	5.1	5	5.1	5.4	6.4	7.1	6.5	4.8	7.1	6.2	5.6	5.4	5.7
Na^+	mmol/L	145	149	149	148	143	154	149	152	145	146	147	145	152	146	149	148	145	148	146	154	144	152	149	149	148	143
$iCa2^+$	mmol/L	1.26	1.28	1.56	1.13	1.24	1.15	1.13	1.24	1.2	1.2	1.28	1.34	1.14	1.22	1.2	1.18	1.38	1.31	1.37	1.38	1.14	1.31	1.35	1.27	1.27	1.21
Glu	mmol/L	4.9	3.7	5.8	7.2	4.6	10.2	4	4.1	3.5	4.3	5.3	4.4	3.4	4.1	4.4	3.1	5.7	4.8	6.7	3.4	3.7	3.1	5.4	3.6	9	4.3
Lac	mmol/L	6.4	3.2	10.1	7.4	5.3	12.4	9.6	8.2	3.8	10.4	8.8	1.3	13.5	2.7	4.4	4.9	9.8	>15	10	>15	8.9	>15	13.5	7.9	>15	>15
nCa^{2+}	mmol/L	1.23	1.27	1.48	1.12	1.23	1.07	1.11	1.12	1.17	1.17	1.26	1.38	1.12	1.17	1.19	1.24	1.32	1.23	1.27	1.22	1.15	1.17	1.22	1.3	1.12	1.17
Hct	%	48	55	>65	54	55	55	60	56	52	59	51	39	54	46	63	50	49	59	54	52	57	59	53	58	5.3	48
SO2	%	95	92	83	98	99	92	37	53	98	99	99	99	99	97	91	99	97	98	94	93	99	70	95	99	75	99
THBC	g/L	163	187	—	184	187	187	204	190	177	201	173	133	184	156	214	170	167	201	184	177	194	201	180	197	180	163

Table S6. Venous blood gas and biochemical analyses in isoflurane anaesthesia (T2) of Sunda pangolins (n_{adult} = 26).

**Animal ID**		MJ-1	MJ-2	MJ-3	MJ-4	MJ-5	MJ-6	MJ-7	MJ-8	MJ-9	MJ-10	MJ-11	MJ-12	MJ-13	MJ-14	MJ-15	MJ-16	MJ-17	MJ-18	MJ-19	MJ-20	MJ-21	MJ-22	MJ-23	MJ-24	MJ-25	MJ-26
Body temperature	℃	33.3	33.3	33.4	33.9	33.1	33.1	33.3	33.4	33	33.6	33.2	33	33.1	33.1	33.9	33.1	33.1	32.7	32.4	32.9	33	33.1	32.9	33	32.5	33.3
H^+	10^{-7} mol/L	5.13	4.37	5.62	5.25	5.37	5.89	3.98	5.75	3.55	6.92	3.89	3.39	5.75	7.08	5.62	3.16	5.01	7.24	5.75	7.24	5.62	5.01	5.13	4.27	7.94	5.89
pH	—	7.35	7.39	7.27	7.38	7.38	7.23	7.35	7.16	7.33	7.34	7.36	7.47	7.36	7.29	7.38	7.52	7.3	7.24	7.22	7.1	7.43	7.12	7.16	7.46	7.09	7.32
PO_2	mmHg	77	63	55	102	126	74	23	37	115	144	139	137	132	96	62	130	95	126	85	90	128	50	95	136	56	129
PCO_2	mmHg	52	62	52	56	44	80	100	109	53	40	49	44	36	59	64	46	45	43	81	96	51	101	80	43	96	46
pHt	—	7.4	7.44	7.32	7.43	7.44	7.28	7.4	7.21	7.39	7.39	7.41	7.53	7.42	7.34	7.43	7.58	7.35	7.3	7.28	7.15	7.49	7.17	7.21	7.52	7.15	7.37
PO_2t	mmHg	60	49	43	85	104	57	18	29	93	125	118	115	110	76	50	108	75	102	63	70	105	38	74	113	41	108
PCO_2t	mmHg	44	53	44	49	37	67	85	93	44	34	41	37	30	50	56	39	38	36	66	80	43	85	67	36	79	39
TCO_2	mmol/L	30.3	39.4	25.5	34.8	27.4	36	58.3	42.2	29.5	22.8	29.2	33.4	21.4	30.2	39.9	39	23.5	19.7	35.7	32.7	35.5	35.9	31	31.9	32	25.1
Beecf	mmol/L	3.1	12.5	-3	8	0.9	5.9	29.6	10.2	2	-4.2	2.3	8.3	-5.1	1.8	12.8	14.7	-4.3	-9	5.5	0.1	9.6	3.5	-0.2	6.8	-0.7	-2.4
BE(B)	mmol/L	1.8	9.1	-3.6	5.7	0.4	2.1	20.4	4.2	0.6	-3.9	1.2	7.4	-4.4	0.2	8.6	12.7	-4.5	-8.8	1.7	-3.7	7.4	-1.6	-3.2	5.8	-4.5	-2.8
$HCO3^-$	mmol/L	28.7	37.5	23.9	33.1	26	33.5	55.2	38.9	27.9	21.6	27.7	32	20.3	28.4	37.9	37.6	22.1	18.4	33.2	29.8	33.9	32.8	28.5	30.6	29.1	23.7
$HCO3^-$ (std)	mmol/L	26.2	31.8	21.7	29.4	25.3	26.3	38.3	26.5	25.4	21.9	25.9	30.7	21.5	25.1	31.3	34.9	21.4	18.1	26.1	21.9	30.7	22.5	22.3	29.5	20.6	22.8
K^+	mmol/L	4.9	5.1	6	5.1	5.7	5.5	4.3	5.5	5.8	5.1	5.7	4.6	5.2	5.1	5	5.1	5.4	6.4	7.1	6.5	4.8	7.1	6.2	5.6	5.4	5.7
Na^+	mmol/L	145	149	149	148	143	154	149	152	145	146	147	145	152	146	149	148	145	148	146	154	144	152	149	149	148	143
$iCa2^+$	mmol/L	1.26	1.28	1.56	1.13	1.24	1.15	1.13	1.24	1.2	1.2	1.28	1.34	1.14	1.22	1.2	1.18	1.38	1.31	1.37	1.38	1.14	1.31	1.35	1.27	1.27	1.21
Glu	mmol/L	4.9	3.7	5.8	7.2	4.6	10.2	4	4.1	3.5	4.3	5.3	4.4	3.4	4.1	4.4	3.1	5.7	4.8	6.7	3.4	3.7	3.1	5.4	3.6	9	4.3
Lac	mmol/L	6.4	3.2	10.1	7.4	5.3	12.4	9.6	8.2	3.8	10.4	8.8	1.3	13.5	2.7	4.4	4.9	9.8	>15	10	>15	8.9	>15	13.5	7.9	>15	>15
nCa^{2+}	mmol/L	1.23	1.27	1.48	1.12	1.23	1.07	1.11	1.12	1.17	1.17	1.26	1.38	1.12	1.17	1.19	1.24	1.32	1.23	1.27	1.22	1.15	1.17	1.22	1.3	1.12	1.17
Hct	%	48	55	>65	54	55	55	60	56	52	59	51	39	54	46	63	50	49	59	54	52	57	59	53	58	5.3	48
SO2	%	95	92	83	98	99	92	37	53	98	99	99	99	99	97	91	99	97	98	94	93	99	70	95	99	75	99
THBC	g/L	163	187	—	184	187	187	204	190	177	201	173	133	184	156	214	170	167	201	184	177	194	201	180	197	180	163
